# High resolution analysis of tropical forest fragmentation and its impact on the global carbon cycle

**DOI:** 10.1038/ncomms14855

**Published:** 2017-03-17

**Authors:** Katharina Brinck, Rico Fischer, Jürgen Groeneveld, Sebastian Lehmann, Mateus Dantas De Paula, Sandro Pütz, Joseph O. Sexton, Danxia Song, Andreas Huth

**Affiliations:** 1 Department of Ecological Modelling, Helmholtz Centre for Environmental Research GmbH-UFZ Leipzig, Permoserstrasse 15, Leipzig 04318, Germany; 2 Department of Mathematics, Imperial College London, South Kensington Campus, London SW7 2AZ, UK; 3 Department of Geographical Sciences, Global Land Cover Facility, University of Maryland, 4321 Hartwick Building, College Park, Maryland 20740, USA; 4 German Centre for Integrative Biodiversity Research (iDiv) Halle-Jena-Leipzig, Leipzig 04103, Germany; 5 Department of Mathematics/Computer Science, Institute for Environmental Systems Research, University of Osnabrück, Osnabrück 49076, Germany

## Abstract

Deforestation in the tropics is not only responsible for direct carbon emissions but also extends the forest edge wherein trees suffer increased mortality. Here we combine high-resolution (30 m) satellite maps of forest cover with estimates of the edge effect and show that 19% of the remaining area of tropical forests lies within 100 m of a forest edge. The tropics house around 50 million forest fragments and the length of the world's tropical forest edges sums to nearly 50 million km. Edge effects in tropical forests have caused an additional 10.3 Gt (2.1–14.4 Gt) of carbon emissions, which translates into 0.34 Gt per year and represents 31% of the currently estimated annual carbon releases due to tropical deforestation. Fragmentation substantially augments carbon emissions from tropical forests and must be taken into account when analysing the role of vegetation in the global carbon cycle.

## Abstract (web summary)

Vast quantities of carbon stored in tropical forests are threatened by deforestation. Here, using high resolution satellite data, Brinck *et al*. examine how edge effects influence carbon emissions and they find an additional 10.3 Gt of carbon are released by deforestation when including fragmentation effects.

Tropical forests are the largest terrestrial component of the global carbon budget^1^,^2^. They account for 50% of the carbon stored in the global vegetation (350–600 Gt C)^3^,^4^,^5^, rivalling the amount of carbon in the atmosphere (750 Gt C)^6^. Carbon losses from tropical forests due to forest loss and degradation are currently estimated to be 1.1 Gt C per year^7^,^8^,^9^. However, quantification of carbon fluxes of tropical forests suffer uncertainties in the measurement of stored biomass^10^,^11^ and rates of forest loss^12^. Moreover, deforestation alters the structure of the remaining forest—a process referred to as fragmentation^13^,^14^,^15^,^16^,^17^,^18^.

Increased wind speed, vapour pressure deficit and disturbance pressure in the vicinity of forest edges increase tree mortality^15^, resulting in persistent carbon emissions long after deforestation occurs. Fire, wind and desiccation can penetrate up to kilometres into the forest^15^,^19^,^20^, altering microclimate within 100–300 m of the forest edge^13^,^21^,^22^,^23^. Field studies have estimated relative carbon losses in forest edges (*e*) averaging 11% (ref. 24) and up to 36% (ref. 22) and 50% (ref. 25) of aboveground biomass within the 100 m edge zone, and modelling studies have shown biomass reductions of up to 70% in small fragments^26^.

Although regional estimates of fragmentation state and carbon losses have been obtained by combining local findings and modelling results^27^, a comprehensive pan-tropical analysis using high-resolution maps is missing. A recent pan-tropical analysis based on coarse-resolution data (500 m) found reductions of biomass extending 1.5 km from the forest edge^20^. Another analysis estimated the distance from all forests to forest edge using remote sensing data^18^.

Here we analysed the extent of tropical forest fragmentation and its effects on the global carbon balance of the terrestrial biosphere. We quantified the total number of forest fragments and the area of their edges across the tropics using a global, high-resolution (30 m) tree-cover data set from the year 2000 (ref. 28). We estimated biomass loss in the edge area based on a biomass reduction factor and biomass values of the forest core areas that we extracted from a published aboveground biomass map^29^. Then we overlaid the high-resolution forest edge analysis with this biomass loss assumption to incorporate the edge effect into the global carbon balance of tropical forests. We find that fragmentation substantially increases carbon emissions from tropical forests by 31% (of the currently estimated annual carbon losses due to tropical deforestation) and is hence relevant for the global carbon cycle.

## Results

### Analysis of tropical forest fragmentation

**Figure 1:**
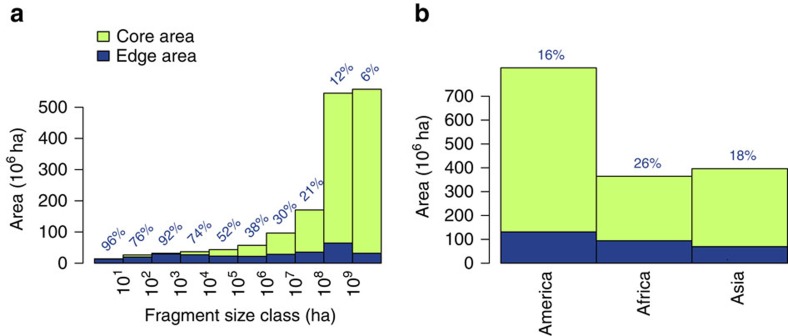
Fragment size distribution for tropical forest. Distribution of total forested area, core area (green) and edge area (blue; edge depth=100 m, blue numbers: edge area/total area (%)) for different fragment size classes (**a**) and different continents (**b**).

The tropics house >50 million forest fragments with a mean fragment area of 29 ha and total area >1.5 billion ha (Table 1). Size–frequency distributions of forest fragments are similar across continents (see Supplementary Fig. 1). The length of the world's tropical forest edges sums to nearly 50 million km, and a total of 295 million ha (19%) of tropical forest area is <100 m from the forest edge. Depending on the complexity of each fragment's shape, the proportion of a fragment's edge area decreases with increasing fragment size (Fig. 1a). In tropical America and Asia, the fraction of edge area lies between 16% and 18% of total forest area and is much lower than in Africa (26%) (Fig. 1b).

Additionally, we found that 84% of the edge area is anthropogenically created (see Supplementary Table 1). In Asia, the ratio of anthropogenic to total edge area is 95%—higher than in Africa (63%) and South America (83%). Only 1% of the total forest-edge area in the tropics represents the tree line (America 1.4%, Africa 0.5%, Asia 0.4%). Analysing how much forest fragments are created due to corridor-like clear cuts (for example, road building), we found that 21% of total edge area has a characteristic similar to a corridor (America 20%, Africa 20%, Asia 22%).

### Impact of tropical fragmentation on the global carbon cycle

**Figure 2:**
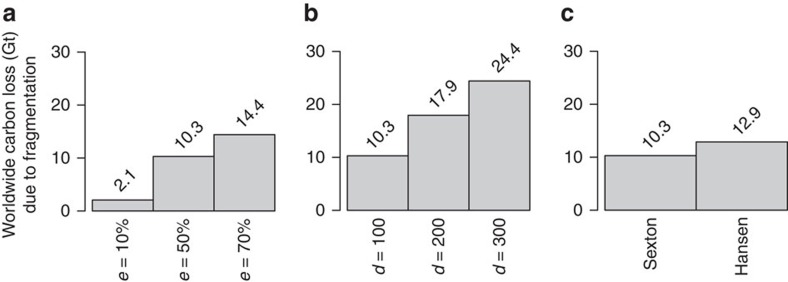
Total carbon losses due to tropical forest fragmentation. Comparing carbon-loss scenarios assuming different assumptions for relative carbon losses *e* in the forest edge area, for different edge penetration distances *d* and different underlying forest cover maps. The numbers represent the total carbon loss due to fragmentation for different scenarios. (**a**) Carbon loss for different assumptions of relative carbon losses in forest edges *e* assuming edge depth *d=*100 m; (**b**) carbon loss for different assumptions of edge depth *d* assuming relative carbon losses in forest edges of *e*=50%; (**c**) comparison of results based on different forest cover maps^28^,^32^ assuming *d=*100 m and *e*=50% (for additional details, see Supplementary Tables 2 and 3).

Aboveground carbon storage of tropical forests worldwide amounts to 161 Gt C, of which 21 Gt C are located within 100 m of the forest edge (Table 1). Assuming edge penetration depth of 100 m (ref. 30) and carbon losses of *e=*50% (refs 25, 26) in the edge areas leads to a release of 10.3 Gt C due to tropical fragmentation (Table 1, Fig. 2a,b). We compared different scenarios of edge depth and fraction of lost biomass to explore the sensitivity of the results. Varying fraction of lost biomass *e* from 10% to 70% (for a 100 m wide edge depth), results in total carbon losses between 2.1 and 14.4 Gt C (Fig. 2a). Comparing carbon-loss scenarios for edge depths from 100 to 300 m while assuming carbon loss of *e=*50% in the edge areas leads to a release of 10.3 to 24.4 Gt C from the fragment edges (Fig. 2b). Carbon-loss estimates are robust to the choice of underlying forest cover map (Fig. 2c). The 30 m resolution forest cover maps from Sexton *et al*.^28^ and Hansen *et al*.^31^ yield similar results for carbon losses from forest fragment edges, with the Sexton data set providing the more conservative estimate (10.3 Gt resp. 12.9 Gt; compare Table 1 and Supplementary Table 2). Vegetation cover maps with lower resolution (GlobCover map^32^ 300 m) give lower estimates for carbon losses (5.42 Gt) as many of the small forest fragments and fine-scale edge structures are neglected (Supplementary Table 1).

**Figure 3:**
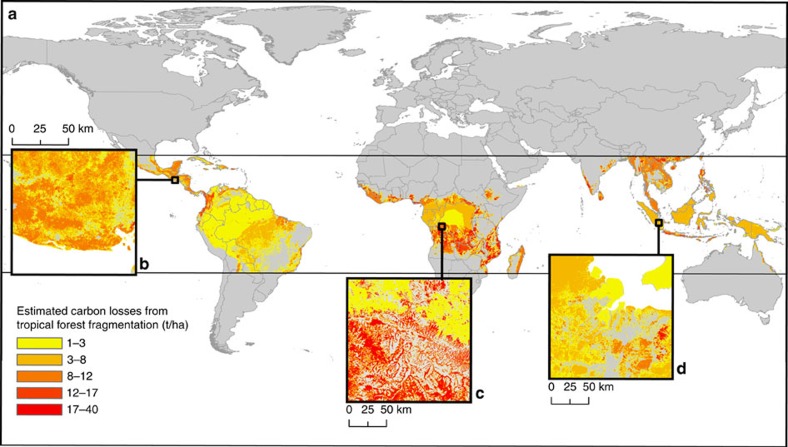
Worldwide carbon emissions due to fragmentation of tropical forests. (**a**) Colours represent the estimated carbon losses for each fragment, setting edge depth *d* to 100 m and relative carbon losses in forest edges *e* to 50%. Insets illustrate exemplary regional carbon emissions for (**b**) tropical America (89.752 W, 13.515 N), (**c**) tropical Africa (17.206 E, 4.499 S) and (**d**) tropical South-East Asia (103.898 E, 3.091 S).

The global distribution of carbon losses from tropical forest fragmentation is shown in Fig. 3. Although the Amazon houses the world's largest area of intact tropical forests, the prevalence of deforestation and fragmentation across Central and South America results in South America leading the world in total carbon emissions. The large, intact forests of the Congo basin are also abutted by highly fragmented forests, leading to more than a fourth of Africa's total forested area lying within 100 m of the forest edge. Asia accounts for the second largest share of carbon emissions among continents (see Table 1), explained by the very high fragmentation due to topography and small-scale fragmentation.

Previous studies estimated a lag of 10–50 years for newly created forest edges to reach new, postdisturbance equilibria^24^,^26^. Assuming that the majority of biomass is lost during the first 30 years^27^ and setting edge depth to 100 m (ref. 30) and *e* to 50% of standing biomass^25^,^26^, the overall 10.3 Gt C lost from fragmentation would contribute 0.34 Gt C per year to the annual global carbon cycle.

## Discussion

**Figure 4:**
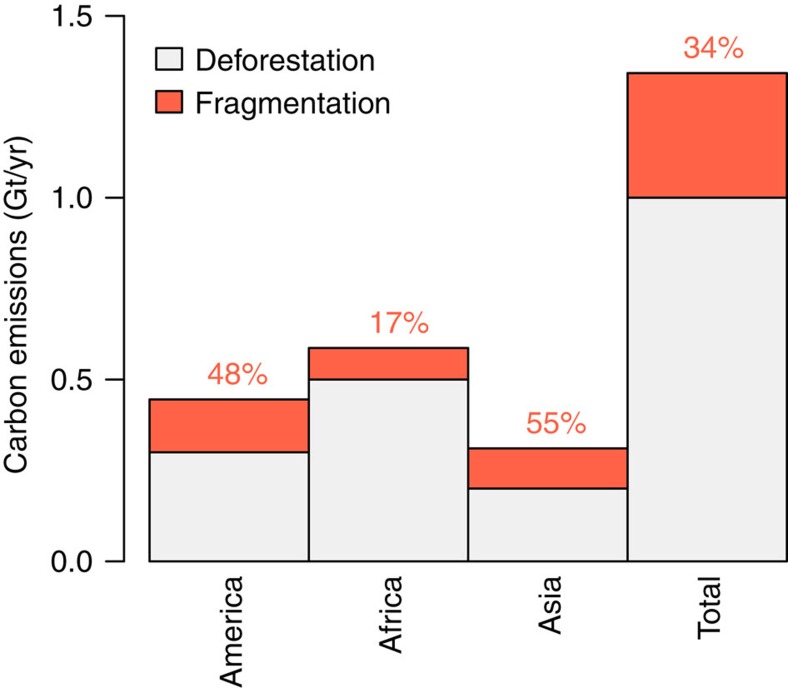
Annual carbon emissions from tropical forests. Annual carbon emissions from tropical deforestation^2^ (grey bars) and additional emissions from tropical forest edges due to fragmentation from this study (orange bars). The percentages represent the fraction of tropical fragmentation carbon emissions and tropical deforestation carbon emissions.

This estimated carbon losses due to tropical forest fragmentation (0.34 Gt C per year) equals 31% of the direct carbon emissions due to land-use changes in tropical forest regions^7^ globally (1.1 Gt C per year) and 8.5% of the overall annual atmospheric carbon gain^5^ (Fig. 4). Whereas many studies have concluded the intact tropical biome are carbon sinks^4^,^33^, if land-use change is considered tropical forests are a carbon source^8^. Our results corroborate the latter and indicate that additional carbon emissions from tropical forest fragmentation increase overall carbon emissions to the atmosphere to be higher values than previously assumed. The estimated absolute carbon losses due to tropical fragmentation (10.3 Gt C) are higher than estimated values in a previous study (6.7 Gt C, Pütz *et al*.^27^) and are within the same order as another recent study based on coarser-resolution maps (9.4 Gt C, 500 m resolution, Chaplin-Kramer *et al*.^20^). The study by Pütz *et al*.^27^ have calculated lower carbon losses compared to this study, as they did a detailed analysis only for South America. However, the fragment counting and forest edge area is different for the continents (Table 1). As fragmentation effects occur on small spatial scales (<100 m), our study benefits from the high (30 m) resolution of the satellite-based tree-cover map, which avoided mixing of forest edges with unaffected forests and other land-cover types within pixels.

Our assumption for the distance of edge-depth penetration (*d*=100 m) and the resulting estimates of carbon loss are conservative; other studies have shown reductions of biomass extending up to 1.5 km from the forest edge^20^. To investigate the effect of the parameter for biomass reduction within the edge area, we investigated the range of lower (*e*=10%) to higher values (*e*=70%) and in our standard assumption *e*=50% (refs 20, 25, 26). Incorporating the shape and edge-length of forest fragments from satellite-based maps overcomes the potential limitation imposed by incorporating only estimates of area and simplified shapes. However, the assumption of the rate of edge creation over time depends on harvesting strategies^12^ and history^34^, so conclusions are challenging with the current state of knowledge and open up further questions in the field of dynamic landscape development. Our analyses show that forest fragmentation augments carbon emissions beyond those caused by deforestation. Although we expect that our study bases on conservative assumptions, they are already substantial enough to highlight the importance of forest fragmentation in the global carbon balance.

## Methods

### Methods summary

Our analysis is based on the GLCF (Global Land Cover Facility) tree-cover data set, a global, 30 m resolution map of fractional tree-canopy cover in the year 2000 (ref. 28). For the analyses of forest patches, we developed a C++ program that implements an adapted Hoshen–Copelman algorithm^35^. Core and edge areas of forest patches were calculated from fragment size and perimeter^36^. Carbon losses were estimated based on a range of biomass-reduction factors and carbon densities of tropical forest from a global aboveground biomass map^29^ by calculating a mean aboveground biomass for each fragment. It was thereby assumed, conservatively, that no root or soil carbon contributes to the emissions from the edge areas. To account for uncertainties in the parameters, scenarios of edge depth and the fraction of carbon loss in edge areas were compared. The influence of the choice of underlying tree-cover data was assessed by comparing results between the GLCF global tree-cover map^28^ (30 m resolution), the Hansen global map of forest cover change^31^ (30 m resolution) and the GlobCover land cover map^32^ (300 m resolution) (Table 1, Supplementary Tables 2 and 3). We also compared results based on different biomass maps, including the biomass map by Saatchi *et al*.^29^ (1,000 m), the biomass map by Baccini *et al*.^2^ (500 m), the biomass map by Avitabile *et al*.^37^ (1,000 m) and mean values from the global biomass data set compiled from Pan *et al*.^4^ (see Supplementary Table 3). The time span after edge creation during which carbon is emitted from the edge areas was taken from previous studies (suggesting 10–50 years^24^,^26^) for estimating annual carbon emission rates. A detailed description of the methods can be found below. The data of the findings (Fig. 3) of this study are available from the corresponding author upon request.

### Forest cover and biomass data

The forest-fragment distribution is based on the global tree-cover map by Sexton *et al*.^28^ for the year 2000. This map has a 30 m spatial resolution. The tree-cover threshold to define forest cover is set to 30%.

To compare the effects of different maps, results based on the Sexton tree-cover map are compared with the Hansen global map of twenty-first century forest cover change^31^ and the GlobCover land cover map^32^. The Hansen global map is based on Landsat data and infers forest-cover loss between 2000 and 2012 at 30 m resolution. Forested areas are defined as having a tree cover of >30%. The GlobCover land cover map (http://due.esrin.esa.int/page_globcover.php) from the year 2009 was produced by the ESA GlobCover 2009 Project (300 m resolution). The land-cover map is based on a time series of global MERIS mosaics for the year 2009 and is classified into 22 land cover classes defined according to the United Nations Land Cover Classification System.

Biomass per hectare is taken from the map of forest carbon stocks in tropical regions by Saatchi *et al*.^29^. For comparison, carbon-loss estimates are also calculated based on the carbon map by Baccini *et al*.^2^, the biomass map by Avitabile *et al*.^37^ and alternatively based on continent-wise average biomass densities derived from forest inventory data from Pan *et al*.^4^ (see Supplementary Table 3).

### Connected-component analysis

The binary forest/non-forest image is considered a graph, in which each forest pixel is a single vertex. The connectivity of a pixel is determined by its four-pixel neighbourhood in cardinal directions, N/S/E/W. A forest pixel is considered an ‘edge' if two pixels along that side are non-forest pixels (two pixels represent a distance of around 60 m near the equator).

A classic two-pass algorithm to determine the connected components is impractical; the entire labelled image would need to be stored in memory. For example, South America (+23.5 N, −23.5 S) would consume about ∼300 GB memory with 64-bit labels. We therefore modified the classic Hoshen–Kopelman algorithm^35^ to estimate the most relevant information in a single pass using a tree structure that consumes about 1 GB of memory. As the image was traversed row by row, 2*P*+1 rows of the image were held in memory (here, *P*=2). After one pass, we have a structure, where every fragment has an entry consisting of its area, edge length and biomass.

The biomass of each pixel was retrieved from the biomass map^29^, with a fall-back to a mean value given by Pan *et al*.^4^ when no valid biomass estimate was available for a given pixel. The different map resolutions were combined via nearest neighbour interpolation.

The size of a pixel in the WGS-84 projection is dependent on its geographical position; sizes were corrected using the Haversine formula^38^. As the evaluation for every pixel would be too costly, we linearly interpolated the area along 256 latitudes.

### Estimation of edge and core area

Which proportion of a forest fragment is affected by edge effects depends largely on the shape of the patch: the higher the perimeter-to-core ratio, the higher the fraction of edge-affected area. We applied the Didham and Ewers core area model^36^ to each fragment separately in order to estimate its amount of core and disturbed edge area, respectively. From the size index


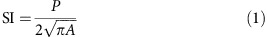


calculated via the fragment's perimeter *P* (m) and its total area *A* (m^2^), the edge-affected area *A*_edge_ (m^2^) is estimated, depending on the depth of the edge effects *d* (m) using the following formula:


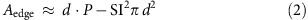


The model is, even for circles, only valid for *d*≤*r*, where *r* is the radius of a circle with the respective size^36^. Therefore, for a given fragment area size *A*, the formula for *A*_edge_ was only used for 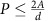 (for a circle if 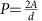, in this case *r* equals *d*) and for 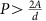 we assume *A*_edge_=*A*. The size index SI is designed such that it yields 1 for circular fragments and larger values for other shapes.

### Estimates of carbon emissions from forest fragment edges

Carbon losses per fragment (t) are calculated as


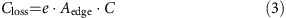


where *e* (%) is the fraction of biomass in the edge area that is lost due to edge effects. *A*_edge_ is the edge-affected area (m^2^) (calculated from equation (2)). *C* (t m^−2^) stands for the amount of carbon stored in the forest (here, 50% of aboveground biomass). Mean aboveground biomass for each fragment were calculated by averaging the biomass values (from the underlying biomass map) for the whole fragment. We assume that this mean biomass values represents potential biomass of the local forest before fragmentation. Additional analyses in Supplementary Discussion 1 confirm that this original analysis (averaging the biomass values) is conservative.

The estimated 0.34 Gt annual carbon losses reflect 31% of the annual carbon emissions due to land-use change in tropical forest regions, based on annual emissions (2000–2009) of 1.1±0.11 Gt C reported by Houghton *et al*.^7^. The annual total carbon flux to the atmosphere is estimated to equal 4 Gt per year (2000–2009)^5^. The carbon emissions due to tropical forest fragmentation found in this study of 0.34 Gt correspond to 8.5% of those total emissions.

### Natural and anthropogenic edges

Natural transition from tropical forest to other biomes (for example, open forests and savannahs) or landscape features such as water bodies most probably have existed since centuries and therefore do not contribute to any additional carbon emissions today, whereas edges joining anthropogenically created land-use covers such as cropland are thought to have once been part of the interior forest. For the GlobCover 2009 map, we distinguish natural edges from non-natural anthropogenically created edges (see Supplementary Table 1). We assume that natural edges occur where tropical forest lies next to moist savannahs (the climatically successive vegetation zone), flooded mangrove forests and the coast, there we expect the vegetation to have already reached an equilibrium state centuries ago and therefore additional carbon losses do not contribute to our balance. Edges are classified as anthropogenic if forest is neighboured by grassland, cropland or artificial surfaces.

Natural edges are defined by a border between tropical forest and one of the following land cover classes: 50: ‘Closed (>40%) broadleaved deciduous forest (>5 m)', 60: ‘Open (15–40%) broadleaved deciduous forest/woodland (>5 m)', 170: ‘Closed (>40%) broadleaved forest or shrubland permanently flooded—saline or brackish water', 180: ‘Closed to open (>15%) grassland or woody vegetation on regularly flooded or waterlogged soil—fresh, brackish or saline water', and 210: ‘Water bodies'.

### Treeline edges

To estimate forest edges that represent the natural tree line, we summed up all forest edges located in regions with elevation >3,000 m according to Körner^39^.

### Corridor edges

We analysed how much non-forest areas have the character of a corridor (for example, due to roads). A forest fragment edge belongs to the corridor class if the distance between two forest fragments is less than two pixels (∼60 m). That means that a forest pixel is followed by two non-forest pixels and then again a forest pixel occurs. The connectivity of a pixel is determined by its four-pixel neighbourhood in cardinal directions, N/S/E/W.

### Data availability

The used input data (global forest cover maps and biomass maps) can be found in the corresponding references. The resulting data set of our analysis—worldwide carbon emissions due to fragmentation of tropical forests with a resolution of 30 m (Fig. 3)—can be requested from the corresponding author (size ∼900 GB). The same map with a coarser resolution of 300 m is downloadable from the following link https://oc.ufz.de/index.php/s/fOvlXYUQqByxJgH (password: fragmentation).

## Additional information

**How to cite this article:** Brinck, K. *et al*. High resolution analysis of tropical forest fragmentation and its impact on the global carbon cycle. *Nat. Commun.* **8**, 14855 doi: 10.1038/ncomms14855 (2017).

**Publisher's note**: Springer Nature remains neutral with regard to jurisdictional claims in published maps and institutional affiliations.

## Supplementary Material

Supplementary Information Supplementary figure, supplementary tables, supplementary discussion and supplementary references

## Figures and Tables

**Table 1:** Forest fragmentation across tropical regions.

	**Unit**	**America**	**Africa**	**Asia**	**Total**
Number of fragments		23,491,573	22,894,239	7,593,226	53,979,038
Total forested area	(10^6^ ha)	819	364	396	1,579
Average fragment size	(ha)	35	16	52	29
Total edge length	(10^6^ km)	22	18	10	50
Total edge area	(10^6^ ha)	131	94	70	295
Total edge area/total forested area	(%)	16	26	18	19
Average aboveground carbon stock	(t C ha^−1^)	101	90	114	102
Aboveground carbon stock in edge area	(Gt C)	8.72	5.20	6.66	20.58
Total aboveground carbon stock	(Gt C)	83	33	45	161
Total carbon losses	(Gt C)	4.36	2.60	3.33	10.29

Fragment statistic was analysed based on the GLCF (Global Land Cover Facility) tree-cover data set^28^ and the biomass map by Saatchi *et al*.^29^ (edge depth *d=*100 m, fraction of carbon loss in the edge area *e*=50%).

## References

[b1] Bonan G. B. Forests and climate change: forcings, feedbacks, and the climate benefits of forests. Science 320, 1444–1449 (2008). 18556546 10.1126/science.1155121

[b2] Baccini A. . Estimated carbon dioxide emissions from tropical deforestation improved by carbon-density maps. Nat. Clim. Chang. 2, 182–185 (2012).

[b3] Houghton R. A., Hall F. & Goetz S. J. Importance of biomass in the global carbon cycle. J. Geophys. Res. 114, G00E03 (2009).

[b4] Pan Y. . A large and persistent carbon sink in the world's forests. Science (80-.) 333, 988–993 (2011). 10.1126/science.1201609 21764754

[b5] Ciais P. . in Climate Change 2013: The Physical Science Basis. Contribution of Working Group I to the Fifth Assessment Report of the Intergovernmental Panel on Climate Change eds Stocker T. F.. 465–570Cambridge University Press (2013).

[b6] Grace J. Understanding and managing the global carbon cycle. J. Ecol. 92, 189–202 (2004).

[b7] Houghton R. A. . Carbon emissions from land use and land-cover change. Biogeosciences 9, 5125–5142 (2012).

[b8] Mahli Y. The carbon balance of tropical forest regions. Curr. Opin. Environ. Sustain. 2, 237–244 (2010).

[b9] Grace J., Mitchard E. & Gloor E. Perturbations in the carbon budget of the tropics. Glob. Ecol. Biogeogr. 20, 3238–3255 (2014). 10.1111/gcb.12600 PMC4261894 24902948

[b10] Goetz S. J. . Measurement and monitoring needs, capabilities and potential for addressing reduced emissions from deforestation and forest degradation under REDD+. Environ. Res. Lett. 10, 123001 (2015).

[b11] Houghton R. A., Lawrence K., Hackler J. & Brown S. The spatial distribution of forest biomass in the Brazilian Amazon: a comparison of estimates. Glob. Chang. Biol. 7, 731–746 (2007).

[b12] Houghton R. A. Aboveground forest biomass and the global carbon balance. Glob. Chang. Biol. 11, 945–958 (2005).

[b13] Laurance W. F. . The fate of Amazonian forest fragments: A 32-year investigation. Biol. Conserv. 144, 56–67 (2011).

[b14] Laurance W. F., Delamonica P., Laurance S. G., Vasconcelos H. L. & Lovejoy T. E. Rainforest fragmentation kills big trees. Nature 404, 836 (2000). 10786782 10.1038/35009032

[b15] Laurance W. F. Do edge effects occur over large spatial scales? Trends Ecol. Evol. 15, 134–135 (2000). 10717681 10.1016/s0169-5347(00)01838-3

[b16] Robinson G. R. . Diverse and contrasting effects of habitat fragmentation. Science (80-.) 257, 524–526 (1992). 10.1126/science.257.5069.524 17778686

[b17] Ewers R. M. . A large-scale forest fragmentation experiment: the Stability of Altered Forest Ecosystems Project. Philos. Trans. R. Soc. Lond. B. Biol. Sci. 366, 3292–3302 (2011). 22006969 10.1098/rstb.2011.0049 PMC3179633

[b18] Haddad N. M. . Habitat fragmentation and its lasting impact on Earth's ecosystems. Sci. Adv. 1, e1500052 (2015). 26601154 10.1126/sciadv.1500052 PMC4643828

[b19] Briant G., Gond V. & Laurance S. G. Habitat fragmentation and the desiccation of forest canopies: a case study from eastern Amazonia. Biol. Conserv. 143, 2763–2769 (2010).

[b20] Chaplin-Kramer R. . Degradation in carbon stocks near tropical forest edges. Nat. Commun. 6, 10158 (2015). 26679749 10.1038/ncomms10158 PMC4703854

[b21] Laurance W. F. Theory meets reality: how habitat fragmentation research has transcended island biogeographic theory. Biol. Conserv. 141, 1731–1744 (2008).

[b22] Laurance W. F. . Biomass collapse in Amazonian forest fragments. Science 278, 1117–1118 (1997).

[b23] Broadbent E. N. . Forest fragmentation and edge effects from deforestation and selective logging in the Brazilian Amazon. Biol. Conserv. 141, 1745–1757 (2008).

[b24] Laurance W. F., Laurance S. G. & Delamonica P. Tropical forest fragmentation and greenhouse gas emissions. For. Ecol. Manage. 110, 173–180 (1998).

[b25] Dantas de Paula M., Alves Costa C. P. & Tabarelli M. Carbon storage in a fragmented landscape of Atlantic forest: the role played by edge-affected habitats and emergent trees. Trop. Conserv. Sci. 4, 349–358 (2011).

[b26] Pütz S., Groeneveld J., Alves L. F., Metzger J. P. & Huth A. Fragmentation drives tropical forest fragments to early successional states: a modelling study for Brazilian Atlantic forests. Ecol. Modell. 222, 1986–1997 (2011).

[b27] Pütz S. . Long-term carbon loss in fragmented Neotropical forests. Nat. Commun 5, 5037 (2014). 25289858 10.1038/ncomms6037

[b28] Sexton J. O. . Global, 30-m resolution continuous fields of tree cover: Landsat-based rescaling of MODIS vegetation continuous fields with lidar-based estimates of error. Int. J. Digit. Earth 6, 427–448 (2013).

[b29] Saatchi S. S. . Benchmark map of forest carbon stocks in tropical regions across three continents. Proc. Natl Acad. Sci. 108, 9899–9904 (2011). 21628575 10.1073/pnas.1019576108 PMC3116381

[b30] Laurance W. F. . Ecosystem decay of amazonian forest fragments: a 22-year investigation. Conserv. Biol. 16, 605–618 (2002).

[b31] Hansen M. C. . High-resolution global maps of 21st-century forest cover change. Science 342, 850–853 (2013). 24233722 10.1126/science.1244693

[b32] Arino O., Ramos P., Jose J., Kalogirou V., Bontemps S. & Defourny P. . *Global Land Cover Map for 2009 (GlobCover 2009)*. doi:10.1594/PANGAEA.787668 (European Space Agency (ESA) & Université catholique de Louvain (UCL), 2012).

[b33] Lewis S. . Increasing carbon storage in intact African tropical forests. Nature 457, 1003–1006 (2009). 19225523 10.1038/nature07771

[b34] Numata I., Cochrane M. A., Souza C. M. Jr & Sales M. H. Carbon emissions from deforestation and forest fragmentation in the Brazilian Amazon. Environ. Res. Lett. 6, 44003 (2011).

[b35] Hoshen J. & Kopelman R. Percolation and cluster distribution. I. Cluster multiple labeling technique and critical concentration algorithm. Phys. Rev. B 14, 3438–3445 (1976).

[b36] Didham R. K. & Ewers R. M. Predicting the impacts of edge effects in fragmented habitats: Laurance and Yensen's core area model revisited. Biol. Conserv. 155, 104–110 (2012).

[b37] Avitabile V. . An integrated pan-tropical biomass map using multiple reference datasets. Glob. Chang. Biol. 22, 1406–1420 (2016). 26499288 10.1111/gcb.13139

[b38] Robusto C. C. The Cosine-Haversine formula. Am. Math. Monthly 64, 38–40 (1957).

[b39] Körner C. A re-assessment of high elevation treeline positions and their explanation. Oecologia 115, 445–459 (1998). 10.1007/s004420050540 28308263

