# Supplementary material for: High resolution analysis of tropical forest fragmentation and its impact on the global carbon cycle
Source: Nat Commun. 2017 Mar 17;8:14855. doi: 10.1038/ncomms14855 (PMC5357863; doi:10.1038/ncomms14855)
Supplement: Supplementary Information — Supplementary figure, supplementary tables, supplementary discussion and supplementary references [file ncomms14855-s1.pdf]

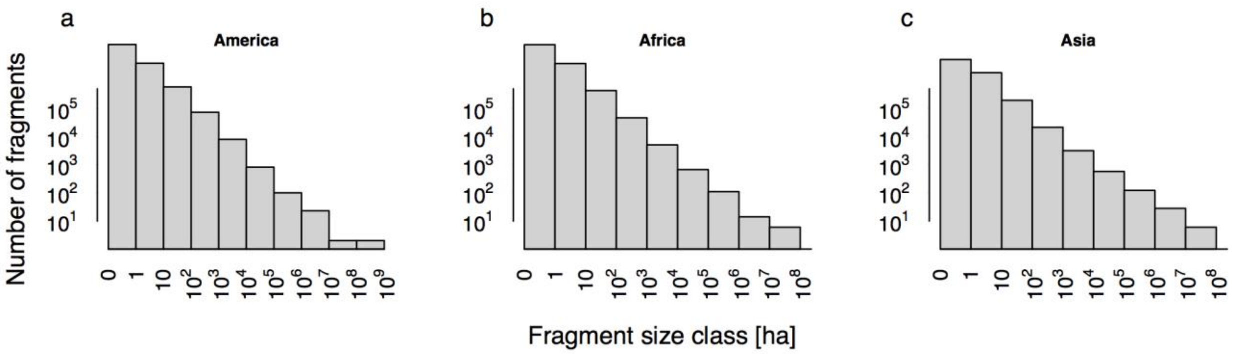

Supplementary Figure 1. Frequency distribution of tropical forest fragment sizes for different continents (a America, b Africa, c Asia). For this study the GLCF (Global Land Cover Facility) tree cover data set[1] with a resolution of 30 m was analyzed. The frequency distribution of forest fragment sizes is similar between continents.

Supplementary Table 1. Analysis of tropical forest fragmentation using GlobCover land cover map and Saatchi's biomass map. Forest fragment statistics for tropical regions (edge depth d = 100 m, fraction of carbon loss in the edge area e = 50%) using the ESA GlobCover land cover map[2] with 300 m resolution and the Saatchi *et al.*[3] biomass map. For details about estimating anthropogenic edge area, see method section 'Natural and Anthropogenic Edges'.

	Unit	America	Africa	Asia	Total
Number of fragments		957,087	459,449	1,005,929	2,422,465
Total forested area	[10^6 ha]	778	229	215	1222
Average fragment size	[ha]	813	498	213	509
Total edge length	[10^6 km]	6	3	5	14
Human induced edge length	[106 km]	4.6	1.7	4.0	10.4
Total edge area	[10^6 ha]	56	26	47	129
Total edge area / total forested area	[%]	7	4	22	11
Anthropogenic edge area / total edge area	[%]	83	63	95	84
Average above-ground carbon stock	[t C/ha]	103	112	121	108
Total above-ground carbon stock	[Gt C]	80	26	26	132
**Total carbon losses**	**[Gt C]**	**1.83**	**1.01**	**2.58**	**5.42**

Supplementary Table 2. Analysis of tropical forest fragmentation using Hansen's forest cover map and Saatchi's biomass map. Forest fragment statistics for tropical regions (edge depth d = 100 m, fraction of carbon loss in the edge area e = 50%) using the Hansen *et al.*[4] forest cover map with 30 m resolution and the Saatchi *et al.*[3] biomass map.

	Unit	America	Africa	Asia	Total
Number of fragments		55,558,018	44,851,251	30,556,204	130,965,473
Total forested area	[10^6 ha]	940	577	391	1908
Average fragment size	[ha]	17	13	13	15
Total edge length	[10^6 km]	34	25	16	75
Total edge area	[10^6 ha]	184	137	94	415
Total edge area / total forested area	[%]	20	24	24	22
Average above-ground carbon stock	[t C/ha]	92	70	114	90
Total above-ground carbon stock	[Gt C]	86	40	45	171
**Total carbon losses**	**[Gt C]**	**4.89**	**3.56**	**4.43**	**12.88**

Supplementary Table 3. Analysis of carbon losses due to tropical forest fragmentation using Sexton's forest cover map and different biomass maps. Carbon losses due to forest fragmentation in tropical regions (edge depth d = 100 m, fraction of carbon loss in the edge area e = 50%) using the Sexton *et al.*[1] 30 m resolution forest cover map and different biomass maps: the Saatchi *et al.* biomass map[3], the Baccini *et al.* biomass map[5], the Avitabile *et al.* biomass map[6], and mean biomass values from Pan *et al.* for intact tropical forest[7]. We got comparable results for carbon losses due to tropical fragmentation for the Saatchi *et al.* biomass map[3] (10.3 Gt), the Avitabile *et al.* biomass map[6] (9.4 Gt) and the Baccini *et al.* biomass map[5] (11.5 Gt), independent of their spatial resolution and their known disagreement in terms of absolute values and spatial distribution of biomass[8]. Estimates based on Pan *et al.*[7] continental carbon stock values lead to higher carbon losses, which underscores the importance of high-resolution data both for forest cover maps and biomass densities.

**Biomass Map**		**Unit**	**America**	**Africa**	**Asia**	**Total**
Saatchi (1000 m)	Average above-ground carbon stock	[t C / ha]	101	90	114	102
	Total above-ground carbon stock	[Gt C]	83	33	45	161
	**Total carbon losses**	**[Gt C]**	**4.36**	**2.60**	**3.33**	**10.29**
Baccini (500 m)	Average above-ground carbon stock	[t C / ha]	116	102	101	109
	Total above-ground carbon stock	[Gt C]	95	37	40	172
	**Total carbon losses**	**[Gt C]**	**5.30**	**3.33**	**2.88**	**11.51**
Avitabile (1000 m)	Average above-ground carbon stock	[t C / ha]	104	108	108	106
	Total above-ground carbon stock	[Gt C]	85	39	42	166
	**Total carbon losses**	**[Gt C]**	**4.03**	**2.51**	**2.85**	**9.39**
Pan (mean values for intact tropical forest)	Average above-ground carbon stock	[t C / ha]	144	116	145	138
	Total above-ground carbon stock	[Gt C]	118	42	57	217
	**Total carbon losses**	**[Gt C]**	**9.42**	**5.44**	**5.05**	**19.91**

Supplementary Discussion 1: Discussion of used approach to calculate biomass losses in forest fragments

We discussed additional analyses to compare different biomass loss assumptions in the edge area of forest fragments (see methods 'Estimates of carbon emissions from tropical forest fragment edges'). Our objective is to estimate the potential biomass loss L_i in fragment i in the edge area with the equation

$$L_i = e \cdot B_i \cdot A_i \ (1),$$

where

B_i: the biomass density of the core area (no edge effects) in fragment i,

A_i: the edge-effected forest area of fragment i,

e: factor for relative biomass losses in forest edge area (e.g., 50%).

The estimation of B_i (biomass density of the core area) is not straightforward. As information about the time since edge creation is not available we estimate $\mathbf{\widehat{B}_i}$ to be the mean biomass of all pixels of fragment i. It is clear that this estimated biomass $\mathbf{\widehat{B}_i}$ will be lower than the "real" value B if the 1 km pixels of the fragment contain not only core area but also edge area (with less biomass) or even non-forested area. Therefore: $\mathbf{\widehat{B}_i} \leq \mathbf{B_i}$. Given that we derive L as mentioned in equation (1). Consequently, the estimated potential biomass losses $\mathbf{\hat{L}_i}$ in the edge area are lower than the real losses L that may already have occurred or that will occur. It follows that:

$$\widehat{B}_i \leq B_i \rightarrow \hat{L}_i \leq L_i \ (2)$$

It means our estimated loss $\mathbf{\hat{L}_i}$ is lower than the loss L that will be caused by fragmentation in the real system.

To assess the effect of the assumption how we estimate B_i, we added an analysis based on two alternative estimates of $\mathbf{\widehat{B}_i}$ (the original analysis is referred as scenario A1 and is documented in the main text in Table 1):

(A2) In this scenario, we take mean biomass values from Pan *et al.*[7], as proxy for biomass density B_i in intact forested core areas. The Pan analysis is based on national forest inventory data. For each continent we used the published mean biomass value for intact tropical forest. Using this approach the carbon losses due to tropical fragmentation are much higher than in the original analysis (A1) presented in the main text:

America: 9.42 Gt C (+116 % compared to results in A1)

Africa: 5.44 Gt C (+109%)

Asia: 5.05 Gt C (+51%)

The results of this analysis are documented in Supplementary Table 3.

(A3) For this scenario we used as proxy for biomass density of the core area B_i the maximum value of all biomass values within on fragment (from Saatchi's biomass map[3]) – assuming that we find the highest biomass values of a fragment in the core area. The total carbon losses due to fragmentation in the tropics are increasing again:

America: 9.86 Gt C (+126% compared to A1)

Africa: 5.9 Gt C (+127%)

Asia: 6.69 Gt C (+101%)

These additional analyses (A2, A3) confirm that our original analysis from the main text (A1) is conservative. More detailed knowledge of the biomass density B_i in undisturbed forested core areas will most likely lead to higher estimates for the biomass loss in edge areas.

Supplementary References

1. Sexton JO, *et al*. Global, 30-m resolution continuous fields of tree cover: Landsat-based rescaling of MODIS vegetation continuous fields with lidar-based estimates of error. *International Journal of Digital Earth* **6**, 427-448 (2013).

2. Arino ORP, J.; Kalogirou, V.; Defourny, P.; Achard, F. GlobCover 2009. In: *ESA Living Planet Symposium*. European Space Agency (2010).

3. Saatchi SS, *et al*. Benchmark map of forest carbon stocks in tropical regions across three continents. *Proceedings of the National Academy of Sciences of the United States of America* **108**, 9899-9904 (2011).

4. Hansen MC, *et al*. High-resolution global maps of 21st-century forest cover change. *Science* **342**, 850-853 (2013).

5. Baccini A, *et al*. Estimated carbon dioxide emissions from tropical deforestation improved by carbon-density maps. *Nat Clim Change* **2**, 182-185 (2012).

6. Avitabile V, *et al*. An integrated pan-tropical biomass map using multiple reference datasets. *Global Change Biology* **22**, 1406-1420 (2016).

7. Pan YD, *et al*. A large and persistent carbon sink in the world's forests. *Science* **333**, 988-993 (2011).

8. Mitchard ETA, *et al*. Uncertainty in the spatial distribution of tropical forest biomass: a comparison of pan-tropical maps. *Carbon Balance and Management* **8**, 1-13 (2013).
